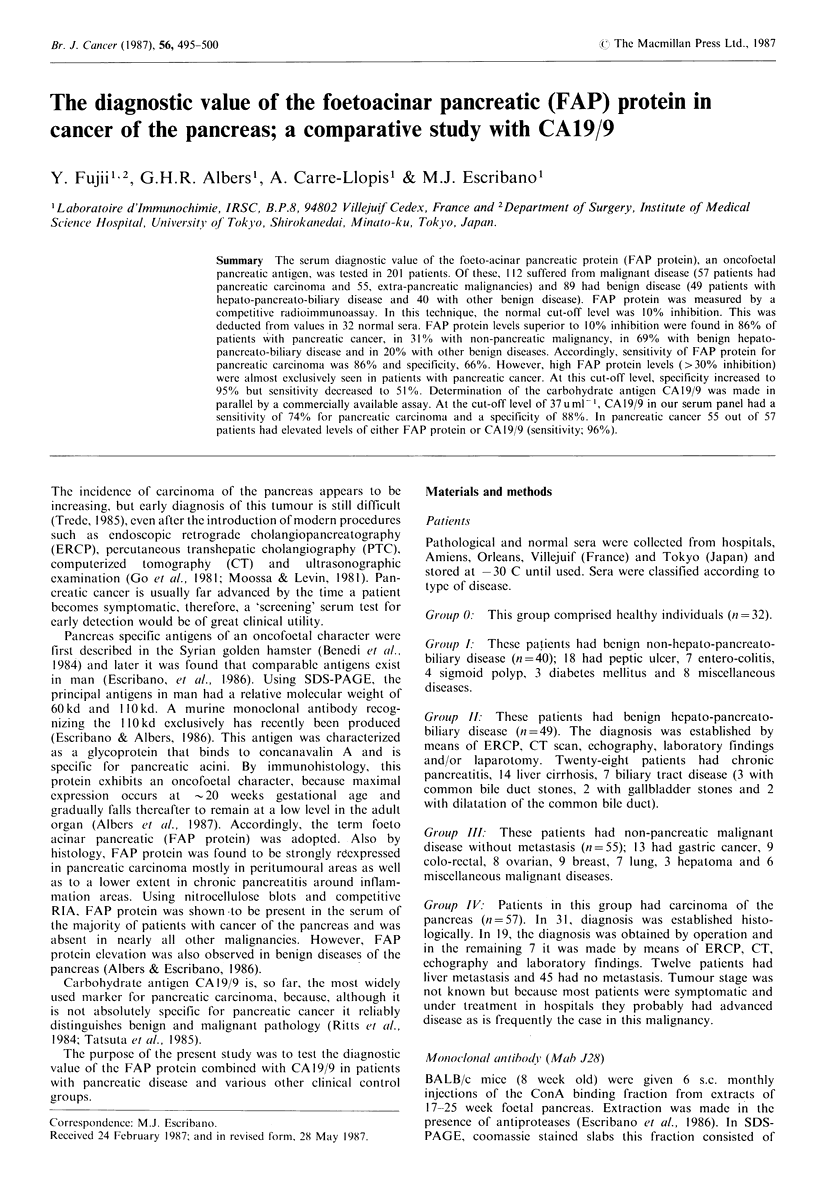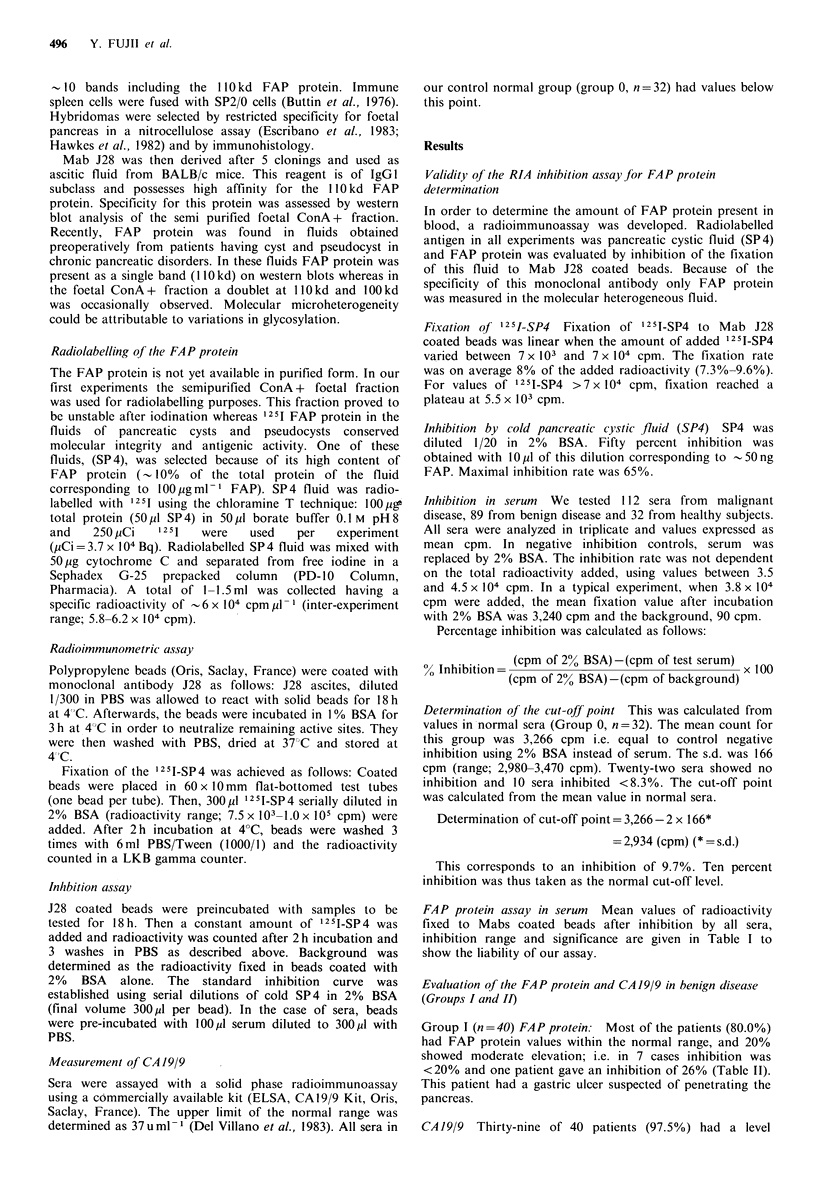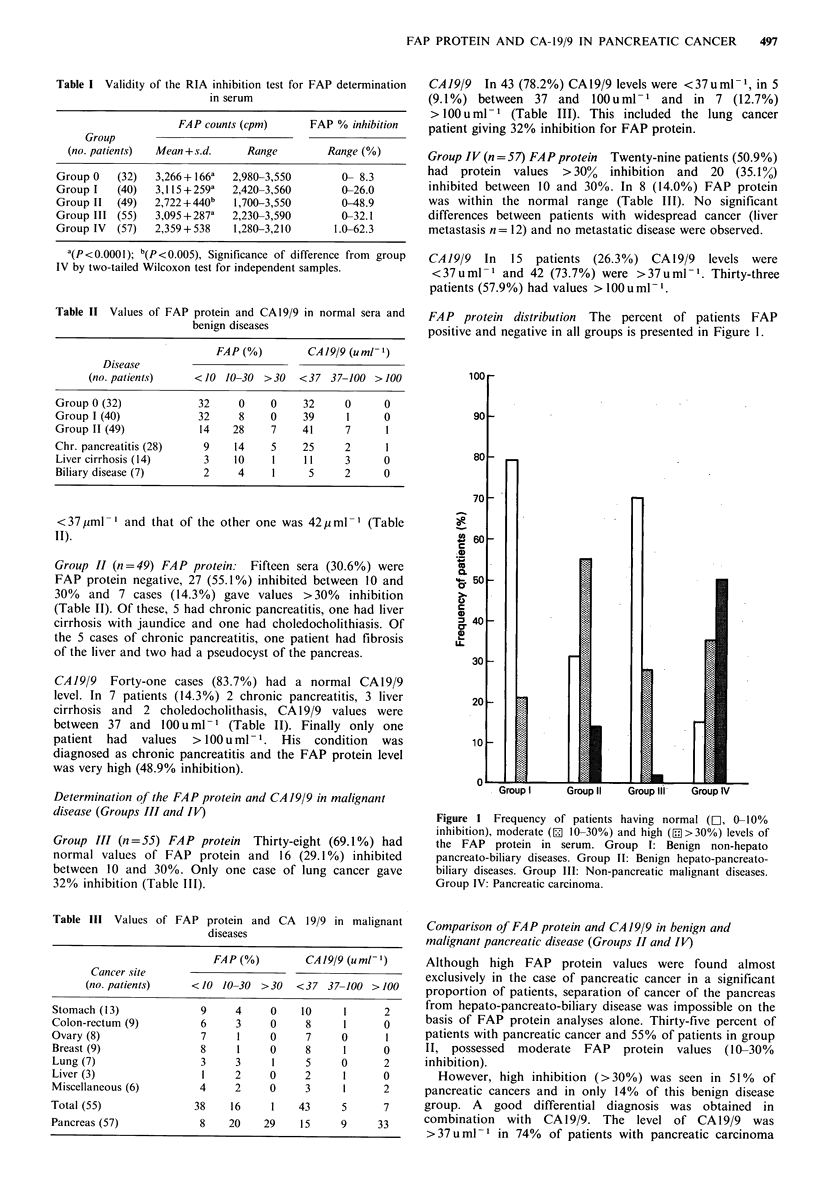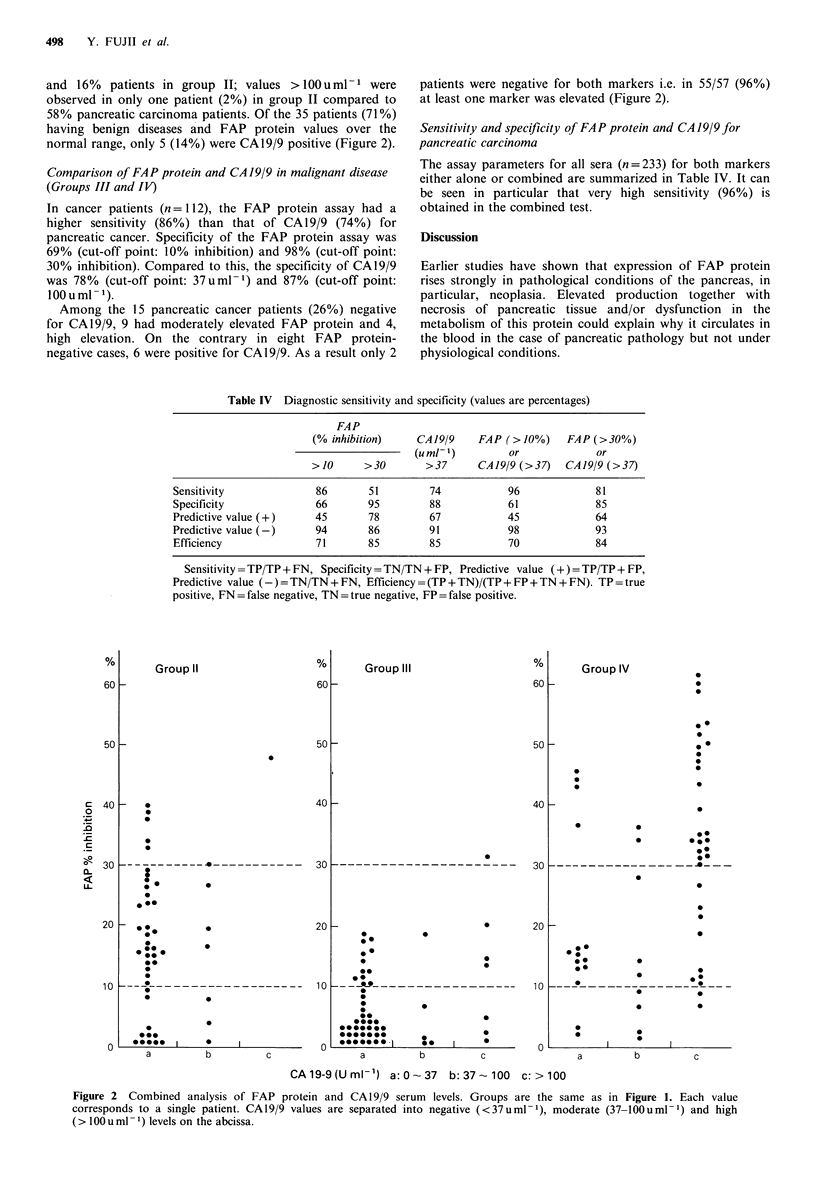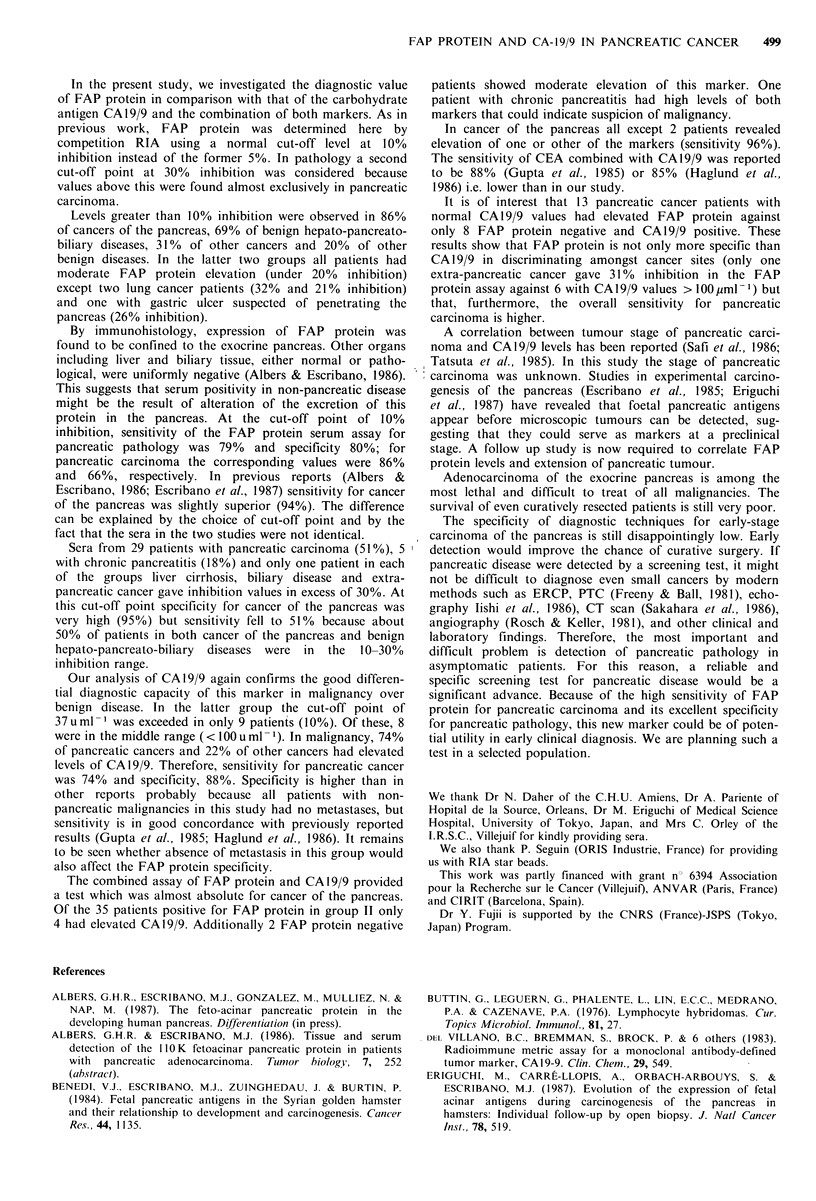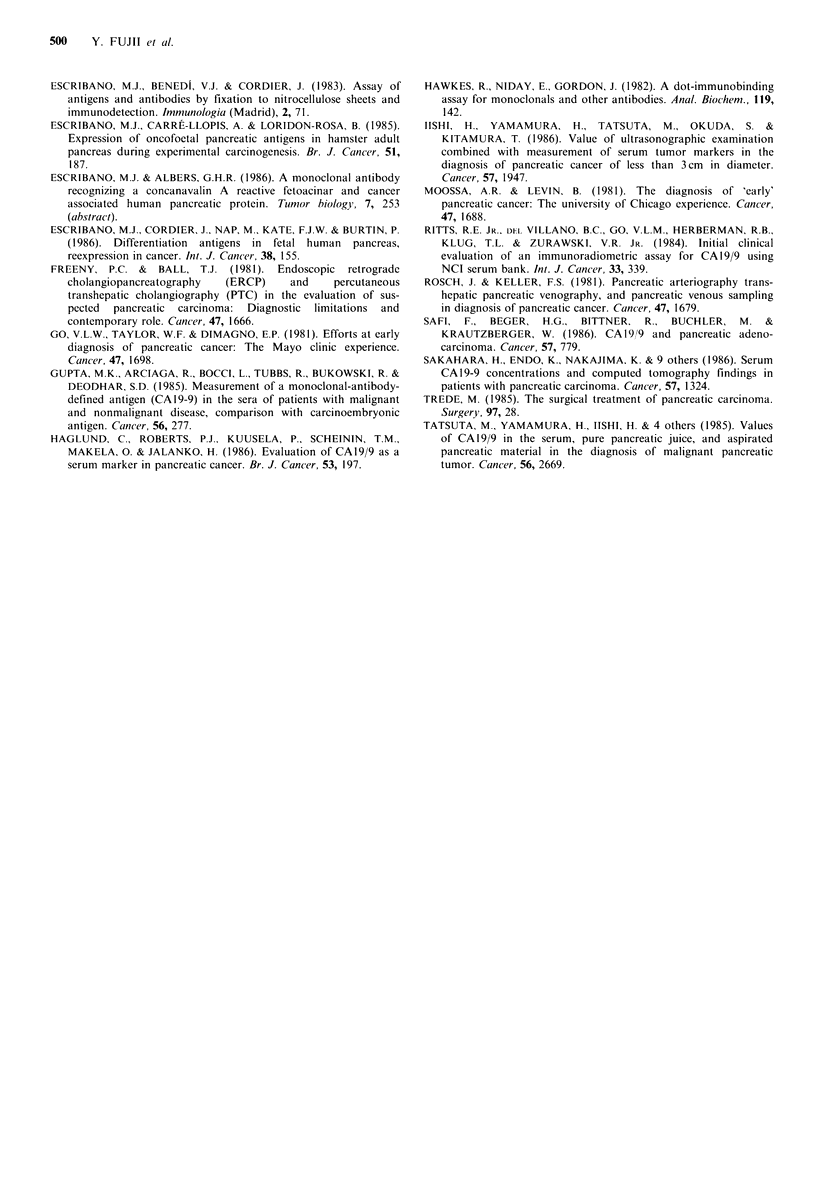# The diagnostic value of the foetoacinar pancreatic (FAP) protein in cancer of the pancreas; a comparative study with CA19/9.

**DOI:** 10.1038/bjc.1987.232

**Published:** 1987-10

**Authors:** Y. Fujii, G. H. Albers, A. Carre-Llopis, M. J. Escribano

**Affiliations:** Laboratoire d'Immunochimie, IRSC, Villejuif, France.

## Abstract

The serum diagnostic value of the foeto-acinar pancreatic protein (FAP protein), an oncofoetal pancreatic antigen, was tested in 201 patients. Of these, 112 suffered from malignant disease (57 patients had pancreatic carcinoma and 55, extra-pancreatic malignancies) and 89 had benign disease (49 patients with hepato-pancreato-biliary disease and 40 with other benign disease). FAP protein was measured by a competitive radioimmunoassay. In this technique, the normal cut-off level was 10% inhibition. This was deducted from values in 32 normal sera. FAP protein levels superior to 10% inhibition were found in 86% of patients with pancreatic cancer, in 31% with non-pancreatic malignancy, in 69% with benign hepato-pancreato-biliary disease and in 20% with other benign diseases. Accordingly, sensitivity of FAP protein for pancreatic carcinoma was 86% and specificity, 66%. However, high FAP protein levels (greater than 30% inhibition) were almost exclusively seen in patients with pancreatic cancer. At this cut-off level, specificity increased to 95% but sensitivity decreased to 51%. Determination of the carbohydrate antigen CA19/9 was made in parallel by a commercially available assay. At the cut-off level of 37 u ml-1, CA19/9 in our serum panel had a sensitivity of 74% for pancreatic carcinoma and a specificity of 88%. In pancreatic cancer 55 out of 57 patients had elevated levels of either FAP protein or CA19/9 (sensitivity; 96%).


Br. J. Cancer (1987), 56, 495–500 © The Macmillan Press Ltd., 1987

### The diagnostic value of the foetoacinar pancreatic (FAP) protein in cancer of the pancreas; a comparative study with CA19/9

Y. Fujii[1,2], G.H.R. Albers[1], A. Carre-Llopis[1] & M.J. Escribano[1]

[1]*Laboratoire d'Immunochimie, IRSC, B.P.8, 94802 Villejuif Cedex, France and* [2]*Department of Surgery, Institute of Medical Science Hospital, University of Tokyo, Shirokanedai, Minato-ku, Tokyo, Japan.*

**Summary** The serum diagnostic value of the foeto-acinar pancreatic protein (FAP protein), an oncofoetal pancreatic antigen, was tested in 201 patients. Of these, 112 suffered from malignant disease (57 patients had pancreatic carcinoma and 55, extra-pancreatic malignancies) and 89 had benign disease (49 patients with hepato-pancreato-biliary disease and 40 with other benign disease). FAP protein was measured by a competitive radioimmunoassay. In this technique, the normal cut-off level was 10% inhibition. This was deducted from values in 32 normal sera. FAP protein levels superior to 10% inhibition were found in 86% of patients with pancreatic cancer, in 31% with non-pancreatic malignancy, in 69% with benign hepato-pancreato-biliary disease and in 20% with other benign diseases. Accordingly, sensitivity of FAP protein for pancreatic carcinoma was 86% and specificity, 66%. However, high FAP protein levels (>30% inhibition) were almost exclusively seen in patients with pancreatic cancer. At this cut-off level, specificity increased to 95% but sensitivity decreased to 51%. Determination of the carbohydrate antigen CA19/9 was made in parallel by a commercially available assay. At the cut-off level of $37\,u\,ml^{-1}$, CA19/9 in our serum panel had a sensitivity of 74% for pancreatic carcinoma and a specificity of 88%. In pancreatic cancer 55 out of 57 patients had elevated levels of either FAP protein or CA19/9 (sensitivity; 96%).

The incidence of carcinoma of the pancreas appears to be increasing, but early diagnosis of this tumour is still difficult (Trede, 1985), even after the introduction of modern procedures such as endoscopic retrograde cholangiopancreatography (ERCP), percutaneous transhepatic cholangiography (PTC), computerized tomography (CT) and ultrasonographic examination (Go *et al.*, 1981; Moossa & Levin, 1981). Pancreatic cancer is usually far advanced by the time a patient becomes symptomatic, therefore, a 'screening' serum test for early detection would be of great clinical utility.

Pancreas specific antigens of an oncofoetal character were first described in the Syrian golden hamster (Benedi *et al.*, 1984) and later it was found that comparable antigens exist in man (Escribano, *et al.*, 1986). Using SDS-PAGE, the principal antigens in man had a relative molecular weight of 60 kd and 110 kd. A murine monoclonal antibody recognizing the 110 kd exclusively has recently been produced (Escribano & Albers, 1986). This antigen was characterized as a glycoprotein that binds to concanavalin A and is specific for pancreatic acini. By immunohistology, this protein exhibits an oncofoetal character, because maximal expression occurs at ~20 weeks gestational age and gradually falls thereafter to remain at a low level in the adult organ (Albers *et al.*, 1987). Accordingly, the term foeto acinar pancreatic (FAP protein) was adopted. Also by histology, FAP protein was found to be strongly reexpressed in pancreatic carcinoma mostly in peritumoural areas as well as to a lower extent in chronic pancreatitis around inflammation areas. Using nitrocellulose blots and competitive RIA, FAP protein was shown to be present in the serum of the majority of patients with cancer of the pancreas and was absent in nearly all other malignancies. However, FAP protein elevation was also observed in benign diseases of the pancreas (Albers & Escribano, 1986).

Carbohydrate antigen CA19/9 is, so far, the most widely used marker for pancreatic carcinoma, because, although it is not absolutely specific for pancreatic cancer it reliably distinguishes benign and malignant pathology (Ritts *et al.*, 1984; Tatsuta *et al.*, 1985).

The purpose of the present study was to test the diagnostic value of the FAP protein combined with CA19/9 in patients with pancreatic disease and various other clinical control groups.

Correspondence: M.J. Escribano.
Received 24 February 1987; and in revised form, 28 May 1987.

### Materials and methods

### *Patients*

Pathological and normal sera were collected from hospitals, Amiens, Orleans, Villejuif (France) and Tokyo (Japan) and stored at −30°C until used. Sera were classified according to type of disease.

*Group 0:* This group comprised healthy individuals ($n=32$).

*Group I:* These patients had benign non-hepato-pancreato-biliary disease ($n=40$); 18 had peptic ulcer, 7 entero-colitis, 4 sigmoid polyp, 3 diabetes mellitus and 8 miscellaneous diseases.

*Group II:* These patients had benign hepato-pancreato-biliary disease ($n=49$). The diagnosis was established by means of ERCP, CT scan, echography, laboratory findings and/or laparotomy. Twenty-eight patients had chronic pancreatitis, 14 liver cirrhosis, 7 biliary tract disease (3 with common bile duct stones, 2 with gallbladder stones and 2 with dilatation of the common bile duct).

*Group III:* These patients had non-pancreatic malignant disease without metastasis ($n=55$); 13 had gastric cancer, 9 colo-rectal, 8 ovarian, 9 breast, 7 lung, 3 hepatoma and 6 miscellaneous malignant diseases.

*Group IV:* Patients in this group had carcinoma of the pancreas ($n=57$). In 31, diagnosis was established histologically. In 19, the diagnosis was obtained by operation and in the remaining 7 it was made by means of ERCP, CT, echography and laboratory findings. Twelve patients had liver metastasis and 45 had no metastasis. Tumour stage was not known but because most patients were symptomatic and under treatment in hospitals they probably had advanced disease as is frequently the case in this malignancy.

### *Monoclonal antibody (Mab J28)*

BALB/c mice (8 week old) were given 6 s.c. monthly injections of the ConA binding fraction from extracts of 17–25 week foetal pancreas. Extraction was made in the presence of antiproteases (Escribano *et al.*, 1986). In SDS-PAGE, coomassie stained slabs this fraction consisted of

~10 bands including the 110 kd FAP protein. Immune spleen cells were fused with SP2/0 cells (Buttin *et al.*, 1976). Hybridomas were selected by restricted specificity for foetal pancreas in a nitrocellulose assay (Escribano *et al.*, 1983; Hawkes *et al.*, 1982) and by immunohistology.

Mab J28 was then derived after 5 clonings and used as ascitic fluid from BALB/c mice. This reagent is of IgG1 subclass and possesses high affinity for the 110 kd FAP protein. Specificity for this protein was assessed by western blot analysis of the semi purified foetal ConA+ fraction. Recently, FAP protein was found in fluids obtained preoperatively from patients having cyst and pseudocyst in chronic pancreatic disorders. In these fluids FAP protein was present as a single band (110 kd) on western blots whereas in the foetal ConA+ fraction a doublet at 110 kd and 100 kd was occasionally observed. Molecular microheterogeneity could be attributable to variations in glycosylation.

*Radiolabelling of the FAP protein*

The FAP protein is not yet available in purified form. In our first experiments the semipurified ConA+ foetal fraction was used for radiolabelling purposes. This fraction proved to be unstable after iodination whereas $^{125}$I FAP protein in the fluids of pancreatic cysts and pseudocysts conserved molecular integrity and antigenic activity. One of these fluids, (SP 4), was selected because of its high content of FAP protein (~10% of the total protein of the fluid corresponding to 100 $\mu$g ml$^{-1}$ FAP). SP 4 fluid was radiolabelled with $^{125}$I using the chloramine T technique: 100 $\mu$g total protein (50 $\mu$l SP 4) in 50 $\mu$l borate buffer 0.1 M pH 8 and 250 $\mu$Ci $^{125}$I were used per experiment ($\mu$Ci = 3.7 × 10$^4$ Bq). Radiolabelled SP 4 fluid was mixed with 50 $\mu$g cytochrome C and separated from free iodine in a Sephadex G-25 prepacked column (PD-10 Column, Pharmacia). A total of 1–1.5 ml was collected having a specific radioactivity of ~6 × 10$^4$ cpm $\mu$l$^{-1}$ (inter-experiment range; 5.8–6.2 × 10$^4$ cpm).

*Radioimmunometric assay*

Polypropylene beads (Oris, Saclay, France) were coated with monoclonal antibody J28 as follows: J28 ascites, diluted 1/300 in PBS was allowed to react with solid beads for 18 h at 4°C. Afterwards, the beads were incubated in 1% BSA for 3 h at 4°C in order to neutralize remaining active sites. They were then washed with PBS, dried at 37°C and stored at 4°C.

Fixation of the $^{125}$I-SP 4 was achieved as follows: Coated beads were placed in 60 × 10 mm flat-bottomed test tubes (one bead per tube). Then, 300 $\mu$l $^{125}$I-SP 4 serially diluted in 2% BSA (radioactivity range; 7.5 × 10$^3$–1.0 × 10$^5$ cpm) were added. After 2 h incubation at 4°C, beads were washed 3 times with 6 ml PBS/Tween (1000/1) and the radioactivity counted in a LKB gamma counter.

*Inhbition assay*

J28 coated beads were preincubated with samples to be tested for 18 h. Then a constant amount of $^{125}$I-SP 4 was added and radioactivity was counted after 2 h incubation and 3 washes in PBS as described above. Background was determined as the radioactivity fixed in beads coated with 2% BSA alone. The standard inhibition curve was established using serial dilutions of cold SP 4 in 2% BSA (final volume 300 $\mu$l per bead). In the case of sera, beads were pre-incubated with 100 $\mu$l serum diluted to 300 $\mu$l with PBS.

*Measurement of CA19/9*

Sera were assayed with a solid phase radioimmunoassay using a commercially available kit (ELSA, CA19/9 Kit, Oris, Saclay, France). The upper limit of the normal range was determined as 37 u ml$^{-1}$ (Del Villano *et al.*, 1983). All sera in our control normal group (group 0, $n$ = 32) had values below this point.

**Results**

*Validity of the RIA inhibition assay for FAP protein determination*

In order to determine the amount of FAP protein present in blood, a radioimmunoassay was developed. Radiolabelled antigen in all experiments was pancreatic cystic fluid (SP 4) and FAP protein was evaluated by inhibition of the fixation of this fluid to Mab J28 coated beads. Because of the specificity of this monoclonal antibody only FAP protein was measured in the molecular heterogeneous fluid.

*Fixation of $^{125}$I-SP4* Fixation of $^{125}$I-SP4 to Mab J28 coated beads was linear when the amount of added $^{125}$I-SP4 varied between 7 × 10$^3$ and 7 × 10$^4$ cpm. The fixation rate was on average 8% of the added radioactivity (7.3%–9.6%). For values of $^{125}$I-SP4 > 7 × 10$^4$ cpm, fixation reached a plateau at 5.5 × 10$^3$ cpm.

*Inhibition by cold pancreatic cystic fluid (SP4)* SP4 was diluted 1/20 in 2% BSA. Fifty percent inhibition was obtained with 10 $\mu$l of this dilution corresponding to ~50 ng FAP. Maximal inhibition rate was 65%.

*Inhibition in serum* We tested 112 sera from malignant disease, 89 from benign disease and 32 from healthy subjects. All sera were analyzed in triplicate and values expressed as mean cpm. In negative inhibition controls, serum was replaced by 2% BSA. The inhibition rate was not dependent on the total radioactivity added, using values between 3.5 and 4.5 × 10$^4$ cpm. In a typical experiment, when 3.8 × 10$^4$ cpm were added, the mean fixation value after incubation with 2% BSA was 3,240 cpm and the background, 90 cpm.

Percentage inhibition was calculated as follows:

$$\%\ \text{Inhibition} = \frac{(\text{cpm of } 2\%\ \text{BSA}) - (\text{cpm of test serum})}{(\text{cpm of } 2\%\ \text{BSA}) - (\text{cpm of background})} \times 100$$

*Determination of the cut-off point* This was calculated from values in normal sera (Group 0, $n$ = 32). The mean count for this group was 3,266 cpm i.e. equal to control negative inhibition using 2% BSA instead of serum. The s.d. was 166 cpm (range; 2,980–3,470 cpm). Twenty-two sera showed no inhibition and 10 sera inhibited < 8.3%. The cut-off point was calculated from the mean value in normal sera.

$$\text{Determination of cut-off point} = 3{,}266 - 2 \times 166^{*}$$
$$= 2{,}934\ (\text{cpm})\ (^{*} = \text{s.d.})$$

This corresponds to an inhibition of 9.7%. Ten percent inhibition was thus taken as the normal cut-off level.

*FAP protein assay in serum* Mean values of radioactivity fixed to Mabs coated beads after inhibition by all sera, inhibition range and significance are given in Table I to show the liability of our assay.

*Evaluation of the FAP protein and CA19/9 in benign disease (Groups I and II)*

Group I ($n$ = 40) *FAP protein:* Most of the patients (80.0%) had FAP protein values within the normal range, and 20% showed moderate elevation; i.e. in 7 cases inhibition was < 20% and one patient gave an inhibition of 26% (Table II). This patient had a gastric ulcer suspected of penetrating the pancreas.

*CA19/9* Thirty-nine of 40 patients (97.5%) had a level

**Table I** Validity of the RIA inhibition test for FAP determination in serum

| Group (no. patients) | FAP counts (cpm) Mean + s.d. | Range | FAP % inhibition Range (%) |
|---|---|---|---|
| Group 0 (32) | 3,266 + 166[a] | 2,980–3,550 | 0– 8.3 |
| Group I (40) | 3,115 + 259[a] | 2,420–3,560 | 0–26.0 |
| Group II (49) | 2,722 + 440[b] | 1,700–3,550 | 0–48.9 |
| Group III (55) | 3,095 + 287[a] | 2,230–3,590 | 0–32.1 |
| Group IV (57) | 2,359 + 538 | 1,280–3,210 | 1.0–62.3 |

[a]($P<0.0001$); [b]($P<0.005$), Significance of difference from group IV by two-tailed Wilcoxon test for independent samples.

**Table II** Values of FAP protein and CA19/9 in normal sera and benign diseases

| Disease (no. patients) | FAP (%) | | | CA19/9 ($u\,ml^{-1}$) | | |
|---|---|---|---|---|---|---|
| | <10 | 10–30 | >30 | <37 | 37–100 | >100 |
| Group 0 (32) | 32 | 0 | 0 | 32 | 0 | 0 |
| Group I (40) | 32 | 8 | 0 | 39 | 1 | 0 |
| Group II (49) | 14 | 28 | 7 | 41 | 7 | 1 |
| Chr. pancreatitis (28) | 9 | 14 | 5 | 25 | 2 | 1 |
| Liver cirrhosis (14) | 3 | 10 | 1 | 11 | 3 | 0 |
| Biliary disease (7) | 2 | 4 | 1 | 5 | 2 | 0 |

<37 $\mu ml^{-1}$ and that of the other one was 42 $\mu ml^{-1}$ (Table II).

*Group II (n = 49) FAP protein:* Fifteen sera (30.6%) were FAP protein negative, 27 (55.1%) inhibited between 10 and 30% and 7 cases (14.3%) gave values >30% inhibition (Table II). Of these, 5 had chronic pancreatitis, one had liver cirrhosis with jaundice and one had choledocholithiasis. Of the 5 cases of chronic pancreatitis, one patient had fibrosis of the liver and two had a pseudocyst of the pancreas.

*CA19/9* Forty-one cases (83.7%) had a normal CA19/9 level. In 7 patients (14.3%) 2 chronic pancreatitis, 3 liver cirrhosis and 2 choledocholithasis, CA19/9 values were between 37 and 100 $u\,ml^{-1}$ (Table II). Finally only one patient had values >100 $u\,ml^{-1}$. His condition was diagnosed as chronic pancreatitis and the FAP protein level was very high (48.9% inhibition).

*Determination of the FAP protein and CA19/9 in malignant disease (Groups III and IV)*

*Group III (n = 55) FAP protein* Thirty-eight (69.1%) had normal values of FAP protein and 16 (29.1%) inhibited between 10 and 30%. Only one case of lung cancer gave 32% inhibition (Table III).

**Table III** Values of FAP protein and CA 19/9 in malignant diseases

| Cancer site (no. patients) | FAP (%) | | | CA19/9 ($u\,ml^{-1}$) | | |
|---|---|---|---|---|---|---|
| | <10 | 10–30 | >30 | <37 | 37–100 | >100 |
| Stomach (13) | 9 | 4 | 0 | 10 | 1 | 2 |
| Colon-rectum (9) | 6 | 3 | 0 | 8 | 1 | 0 |
| Ovary (8) | 7 | 1 | 0 | 7 | 0 | 1 |
| Breast (9) | 8 | 1 | 0 | 8 | 1 | 0 |
| Lung (7) | 3 | 3 | 1 | 5 | 0 | 2 |
| Liver (3) | 1 | 2 | 0 | 2 | 1 | 0 |
| Miscellaneous (6) | 4 | 2 | 0 | 3 | 1 | 2 |
| Total (55) | 38 | 16 | 1 | 43 | 5 | 7 |
| Pancreas (57) | 8 | 20 | 29 | 15 | 9 | 33 |

*CA19/9* In 43 (78.2%) CA19/9 levels were <37 $u\,ml^{-1}$, in 5 (9.1%) between 37 and 100 $u\,ml^{-1}$ and in 7 (12.7%) >100 $u\,ml^{-1}$ (Table III). This included the lung cancer patient giving 32% inhibition for FAP protein.

*Group IV (n = 57) FAP protein* Twenty-nine patients (50.9%) had protein values >30% inhibition and 20 (35.1%) inhibited between 10 and 30%. In 8 (14.0%) FAP protein was within the normal range (Table III). No significant differences between patients with widespread cancer (liver metastasis $n = 12$) and no metastatic disease were observed.

*CA19/9* In 15 patients (26.3%) CA19/9 levels were <37 $u\,ml^{-1}$ and 42 (73.7%) were >37 $u\,ml^{-1}$. Thirty-three patients (57.9%) had values >100 $u\,ml^{-1}$.

*FAP protein distribution* The percent of patients FAP positive and negative in all groups is presented in Figure 1.

**Figure 1** Frequency of patients having normal (□, 0–10% inhibition), moderate (▦ 10–30%) and high (▦ >30%) levels of the FAP protein in serum. Group I: Benign non-hepato pancreato-biliary diseases. Group II: Benign hepato-pancreato-biliary diseases. Group III: Non-pancreatic malignant diseases. Group IV: Pancreatic carcinoma.

*Comparison of FAP protein and CA19/9 in benign and malignant pancreatic disease (Groups II and IV)*

Although high FAP protein values were found almost exclusively in the case of pancreatic cancer in a significant proportion of patients, separation of cancer of the pancreas from hepato-pancreato-biliary disease was impossible on the basis of FAP protein analyses alone. Thirty-five percent of patients with pancreatic cancer and 55% of patients in group II, possessed moderate FAP protein values (10–30% inhibition).

However, high inhibition (>30%) was seen in 51% of pancreatic cancers and in only 14% of this benign disease group. A good differential diagnosis was obtained in combination with CA19/9. The level of CA19/9 was >37 $u\,ml^{-1}$ in 74% of patients with pancreatic carcinoma and 16% patients in group II; values $>100\,u\,ml^{-1}$ were observed in only one patient (2%) in group II compared to 58% pancreatic carcinoma patients. Of the 35 patients (71%) having benign diseases and FAP protein values over the normal range, only 5 (14%) were CA19/9 positive (Figure 2).

*Comparison of FAP protein and CA19/9 in malignant disease (Groups III and IV)*

In cancer patients ($n=112$), the FAP protein assay had a higher sensitivity (86%) than that of CA19/9 (74%) for pancreatic cancer. Specificity of the FAP protein assay was 69% (cut-off point: 10% inhibition) and 98% (cut-off point: 30% inhibition). Compared to this, the specificity of CA19/9 was 78% (cut-off point: $37\,u\,ml^{-1}$) and 87% (cut-off point: $100\,u\,ml^{-1}$).

Among the 15 pancreatic cancer patients (26%) negative for CA19/9, 9 had moderately elevated FAP protein and 4, high elevation. On the contrary in eight FAP protein-negative cases, 6 were positive for CA19/9. As a result only 2 patients were negative for both markers i.e. in 55/57 (96%) at least one marker was elevated (Figure 2).

*Sensitivity and specificity of FAP protein and CA19/9 for pancreatic carcinoma*

The assay parameters for all sera ($n=233$) for both markers either alone or combined are summarized in Table IV. It can be seen in particular that very high sensitivity (96%) is obtained in the combined test.

### Discussion

Earlier studies have shown that expression of FAP protein rises strongly in pathological conditions of the pancreas, in particular, neoplasia. Elevated production together with necrosis of pancreatic tissue and/or dysfunction in the metabolism of this protein could explain why it circulates in the blood in the case of pancreatic pathology but not under physiological conditions.

**Table IV** Diagnostic sensitivity and specificity (values are percentages)

| | *FAP (% inhibition)* | | *CA19/9* ($u\,ml^{-1}$) | *FAP (>10%) or CA19/9 (>37)* | *FAP (>30%) or CA19/9 (>37)* |
|---|---|---|---|---|---|
| | *>10* | *>30* | *>37* | | |
| Sensitivity | 86 | 51 | 74 | 96 | 81 |
| Specificity | 66 | 95 | 88 | 61 | 85 |
| Predictive value (+) | 45 | 78 | 67 | 45 | 64 |
| Predictive value (−) | 94 | 86 | 91 | 98 | 93 |
| Efficiency | 71 | 85 | 85 | 70 | 84 |

Sensitivity = TP/TP + FN, Specificity = TN/TN + FP, Predictive value (+) = TP/TP + FP, Predictive value (−) = TN/TN + FN, Efficiency = (TP + TN)/(TP + FP + TN + FN). TP = true positive, FN = false negative, TN = true negative, FP = false positive.

**Figure 2** Combined analysis of FAP protein and CA19/9 serum levels. Groups are the same as in **Figure 1.** Each value corresponds to a single patient. CA19/9 values are separated into negative ($<37\,u\,ml^{-1}$), moderate ($37$–$100\,u\,ml^{-1}$) and high ($>100\,u\,ml^{-1}$) levels on the abcissa.

In the present study, we investigated the diagnostic value of FAP protein in comparison with that of the carbohydrate antigen CA19/9 and the combination of both markers. As in previous work, FAP protein was determined here by competition RIA using a normal cut-off level at 10% inhibition instead of the former 5%. In pathology a second cut-off point at 30% inhibition was considered because values above this were found almost exclusively in pancreatic carcinoma.

Levels greater than 10% inhibition were observed in 86% of cancers of the pancreas, 69% of benign hepato-pancreato-biliary diseases, 31% of other cancers and 20% of other benign diseases. In the latter two groups all patients had moderate FAP protein elevation (under 20% inhibition) except two lung cancer patients (32% and 21% inhibition) and one with gastric ulcer suspected of penetrating the pancreas (26% inhibition).

By immunohistology, expression of FAP protein was found to be confined to the exocrine pancreas. Other organs including liver and biliary tissue, either normal or pathological, were uniformly negative (Albers & Escribano, 1986). This suggests that serum positivity in non-pancreatic disease might be the result of alteration of the excretion of this protein in the pancreas. At the cut-off point of 10% inhibition, sensitivity of the FAP protein serum assay for pancreatic pathology was 79% and specificity 80%; for pancreatic carcinoma the corresponding values were 86% and 66%, respectively. In previous reports (Albers & Escribano, 1986; Escribano *et al.*, 1987) sensitivity for cancer of the pancreas was slightly superior (94%). The difference can be explained by the choice of cut-off point and by the fact that the sera in the two studies were not identical.

Sera from 29 patients with pancreatic carcinoma (51%), 5 with chronic pancreatitis (18%) and only one patient in each of the groups liver cirrhosis, biliary disease and extra-pancreatic cancer gave inhibition values in excess of 30%. At this cut-off point specificity for cancer of the pancreas was very high (95%) but sensitivity fell to 51% because about 50% of patients in both cancer of the pancreas and benign hepato-pancreato-biliary diseases were in the 10–30% inhibition range.

Our analysis of CA19/9 again confirms the good differential diagnostic capacity of this marker in malignancy over benign disease. In the latter group the cut-off point of $37\,u\,ml^{-1}$ was exceeded in only 9 patients (10%). Of these, 8 were in the middle range ($<100\,u\,ml^{-1}$). In malignancy, 74% of pancreatic cancers and 22% of other cancers had elevated levels of CA19/9. Therefore, sensitivity for pancreatic cancer was 74% and specificity, 88%. Specificity is higher than in other reports probably because all patients with non-pancreatic malignancies in this study had no metastases, but sensitivity is in good concordance with previously reported results (Gupta *et al.*, 1985; Haglund *et al.*, 1986). It remains to be seen whether absence of metastasis in this group would also affect the FAP protein specificity.

The combined assay of FAP protein and CA19/9 provided a test which was almost absolute for cancer of the pancreas. Of the 35 patients positive for FAP protein in group II only 4 had elevated CA19/9. Additionally 2 FAP protein negative patients showed moderate elevation of this marker. One patient with chronic pancreatitis had high levels of both markers that could indicate suspicion of malignancy.

In cancer of the pancreas all except 2 patients revealed elevation of one or other of the markers (sensitivity 96%). The sensitivity of CEA combined with CA19/9 was reported to be 88% (Gupta *et al.*, 1985) or 85% (Haglund *et al.*, 1986) i.e. lower than in our study.

It is of interest that 13 pancreatic cancer patients with normal CA19/9 values had elevated FAP protein against only 8 FAP protein negative and CA19/9 positive. These results show that FAP protein is not only more specific than CA19/9 in discriminating amongst cancer sites (only one extra-pancreatic cancer gave 31% inhibition in the FAP protein assay against 6 with CA19/9 values $>100\,\mu ml^{-1}$) but that, furthermore, the overall sensitivity for pancreatic carcinoma is higher.

A correlation between tumour stage of pancreatic carcinoma and CA19/9 levels has been reported (Safi *et al.*, 1986; Tatsuta *et al.*, 1985). In this study the stage of pancreatic carcinoma was unknown. Studies in experimental carcinogenesis of the pancreas (Escribano *et al.*, 1985; Eriguchi *et al.*, 1987) have revealed that foetal pancreatic antigens appear before microscopic tumours can be detected, suggesting that they could serve as markers at a preclinical stage. A follow up study is now required to correlate FAP protein levels and extension of pancreatic tumour.

Adenocarcinoma of the exocrine pancreas is among the most lethal and difficult to treat of all malignancies. The survival of even curatively resected patients is still very poor.

The specificity of diagnostic techniques for early-stage carcinoma of the pancreas is still disappointingly low. Early detection would improve the chance of curative surgery. If pancreatic disease were detected by a screening test, it might not be difficult to diagnose even small cancers by modern methods such as ERCP, PTC (Freeny & Ball, 1981), echography Iishi *et al.*, 1986), CT scan (Sakahara *et al.*, 1986), angiography (Rosch & Keller, 1981), and other clinical and laboratory findings. Therefore, the most important and difficult problem is detection of pancreatic pathology in asymptomatic patients. For this reason, a reliable and specific screening test for pancreatic disease would be a significant advance. Because of the high sensitivity of FAP protein for pancreatic carcinoma and its excellent specificity for pancreatic pathology, this new marker could be of potential utility in early clinical diagnosis. We are planning such a test in a selected population.

We thank Dr N. Daher of the C.H.U. Amiens, Dr A. Pariente of Hopital de la Source, Orleans, Dr M. Eriguchi of Medical Science Hospital, University of Tokyo, Japan, and Mrs C. Orley of the I.R.S.C., Villejuif for kindly providing sera.

We also thank P. Seguin (ORIS Industrie, France) for providing us with RIA star beads.

This work was partly financed with grant n° 6394 Association pour la Recherche sur le Cancer (Villejuif), ANVAR (Paris, France) and CIRIT (Barcelona, Spain).

Dr Y. Fujii is supported by the CNRS (France)-JSPS (Tokyo, Japan) Program.

### References

ALBERS, G.H.R., ESCRIBANO, M.J., GONZALEZ, M., MULLIEZ, N. & NAP, M. (1987). The feto-acinar pancreatic protein in the developing human pancreas. *Differentiation* (in press).

ALBERS, G.H.R. & ESCRIBANO, M.J. (1986). Tissue and serum detection of the 110K fetoacinar pancreatic protein in patients with pancreatic adenocarcinoma. *Tumor biology*, **7**, 252 (*abstract*).

BENEDI, V.J., ESCRIBANO, M.J., ZUINGHEDAU, J. & BURTIN, P. (1984). Fetal pancreatic antigens in the Syrian golden hamster and their relationship to development and carcinogenesis. *Cancer Res.*, **44**, 1135.

BUTTIN, G., LEGUERN, G., PHALENTE, L., LIN, E.C.C., MEDRANO, P.A. & CAZENAVE, P.A. (1976). Lymphocyte hybridomas. *Cur. Topics Microbiol. Immunol.*, **81**, 27.

DEL VILLANO, B.C., BREMMAN, S., BROCK, P. & 6 others (1983). Radioimmune metric assay for a monoclonal antibody-defined tumor marker, CA19-9. *Clin. Chem.*, **29**, 549.

ERIGUCHI, M., CARRÉ-LLOPIS, A., ORBACH-ARBOUYS, S. & ESCRIBANO, M.J. (1987). Evolution of the expression of fetal acinar antigens during carcinogenesis of the pancreas in hamsters: Individual follow-up by open biopsy. *J. Natl Cancer Inst.*, **78**, 519.

ESCRIBANO, M.J., BENEDÍ, V.J. & CORDIER, J. (1983). Assay of antigens and antibodies by fixation to nitrocellulose sheets and immunodetection. *Immunologia* (Madrid), **2,** 71.

ESCRIBANO, M.J., CARRÉ-LLOPIS, A. & LORIDON-ROSA, B. (1985). Expression of oncofoetal pancreatic antigens in hamster adult pancreas during experimental carcinogenesis. *Br. J. Cancer,* **51,** 187.

ESCRIBANO, M.J. & ALBERS, G.H.R. (1986). A monoclonal antibody recognizing a concanavalin A reactive fetoacinar and cancer associated human pancreatic protein. *Tumor biology,* **7,** 253 (*abstract*).

ESCRIBANO, M.J., CORDIER, J., NAP, M., KATE, F.J.W. & BURTIN, P. (1986). Differentiation antigens in fetal human pancreas, reexpression in cancer. *Int. J. Cancer,* **38,** 155.

FREENY, P.C. & BALL, T.J. (1981). Endoscopic retrograde cholangiopancreatography (ERCP) and percutaneous transhepatic cholangiography (PTC) in the evaluation of suspected pancreatic carcinoma: Diagnostic limitations and contemporary role. *Cancer,* **47,** 1666.

GO, V.L.W., TAYLOR, W.F. & DIMAGNO, E.P. (1981). Efforts at early diagnosis of pancreatic cancer: The Mayo clinic experience. *Cancer,* **47,** 1698.

GUPTA, M.K., ARCIAGA, R., BOCCI, L., TUBBS, R., BUKOWSKI, R. & DEODHAR, S.D. (1985). Measurement of a monoclonal-antibody-defined antigen (CA19-9) in the sera of patients with malignant and nonmalignant disease, comparison with carcinoembryonic antigen. *Cancer,* **56,** 277.

HAGLUND, C., ROBERTS, P.J., KUUSELA, P., SCHEININ, T.M., MAKELA, O. & JALANKO, H. (1986). Evaluation of CA19/9 as a serum marker in pancreatic cancer. *Br. J. Cancer,* **53,** 197.

HAWKES, R., NIDAY, E., GORDON, J. (1982). A dot-immunobinding assay for monoclonals and other antibodies. *Anal. Biochem.,* **119,** 142.

IISHI, H., YAMAMURA, H., TATSUTA, M., OKUDA, S. & KITAMURA, T. (1986). Value of ultrasonographic examination combined with measurement of serum tumor markers in the diagnosis of pancreatic cancer of less than 3 cm in diameter. *Cancer,* **57,** 1947.

MOOSSA, A.R. & LEVIN, B. (1981). The diagnosis of 'early' pancreatic cancer: The university of Chicago experience. *Cancer,* **47,** 1688.

RITTS, R.E. JR., DEL VILLANO, B.C., GO, V.L.M., HERBERMAN, R.B., KLUG, T.L. & ZURAWSKI, V.R. JR. (1984). Initial clinical evaluation of an immunoradiometric assay for CA19/9 using NCI serum bank. *Int. J. Cancer,* **33,** 339.

ROSCH, J. & KELLER, F.S. (1981). Pancreatic arteriography transhepatic pancreatic venography, and pancreatic venous sampling in diagnosis of pancreatic cancer. *Cancer,* **47,** 1679.

SAFI, F., BEGER, H.G., BITTNER, R., BUCHLER, M. & KRAUTZBERGER, W. (1986). CA19/9 and pancreatic adenocarcinoma. *Cancer,* **57,** 779.

SAKAHARA, H., ENDO, K., NAKAJIMA, K. & 9 others (1986). Serum CA19-9 concentrations and computed tomography findings in patients with pancreatic carcinoma. *Cancer,* **57,** 1324.

TREDE, M. (1985). The surgical treatment of pancreatic carcinoma. *Surgery,* **97,** 28.

TATSUTA, M., YAMAMURA, H., IISHI, H. & 4 others (1985). Values of CA19/9 in the serum, pure pancreatic juice, and aspirated pancreatic material in the diagnosis of malignant pancreatic tumor. *Cancer,* **56,** 2669.